# Stable Zinc-Based Metal-Organic Framework Photocatalyst for Effective Visible-Light-Driven Hydrogen Production

**DOI:** 10.3390/molecules27061917

**Published:** 2022-03-16

**Authors:** Li-Long Dang, Ting-Ting Zhang, Ting-Ting Li, Tian Chen, Ying Zhao, Chen-Chen Zhao, Lu-Fang Ma

**Affiliations:** 1 Henan Province Function-Oriented Porous Materials Key Laboratory, College of Chemistry and Chemical Engineering, Luoyang Normal University, Luoyang 471934, China; danglilong8@163.com (L.-L.D.); zhangtingting131@126.com (T.-T.Z.); tingtinglichem@163.com (T.-T.L.); ctian2022@163.com (T.C.); zhaoyingchem@126.com (Y.Z.); chenchenzhao2022@163.com (C.-C.Z.); 2 College of Materials and Chemical Engineering, China Three Gorges University, Yichang 443002, China; 3 Shanghai Key Laboratory of Molecular Catalysis and Innovative Materials, Fudan University, Shanghai 200438, China

**Keywords:** metal-organic framework, photocatalytic hydrogen generation, fluorescence emission

## Abstract

Herein, a new Zn-MOF material, [Zn(**L1**)(**L2**)], **1**, was built successfully through a one-pot solvothermal method. The 3D MOF structure was determined by Single X-ray diffraction analysis, IR, and elemental analysis. A series of PXRD tests of **1** after being immersed in different solvents and pH solutions demonstrated the good stability of **1**. Interestingly, this material displayed high catalytic activity for the visible-light-driven hydrogen generation under the illumination of white LED in pure water or a mixture of DMF and H_2_O without additional photosensitizers and cocatalysts. Besides, the studies also showed that the catalytic activity changed constantly as well as the solvent ratio adjustment of DMF and H_2_O from 4:6 to 2:8. Additionally, the catalytic activity reached the best value (743 μmol g^−1^ h^−1^) when the solvent ratio was 4:6. The heterogeneous nature and recyclability of the MOF catalyst, as well as several factors that affect the catalytic activity, were investigated and described in detail. Moreover, the photocatalytic mechanism for the hydrogen generation of **1** was also proposed based on the fluorescence spectra and UV-vis absorption.

## 1. Introduction

The relentless consumption of fossil fuels raises an urgent issue about how to develop new energy sources to fill the gap of existing energy shortage. In this context, scientists have focused their attention to hydrogen fuel because hydrogen can be produced by water decomposition [1,2,3,4]. Therefore, exploring a variety of materials to facilitate the decomposition of water to generate hydrogen has sparked a scientific boom. During last decade, great progress has been made on splitting water to generate hydrogen covering the catalyst type from the initial homogeneous catalysts to heterogeneous species [5,6,7,8], improvement of catalytic performance, and repeatability optimization of the catalysts. The heterogeneous catalysts are increasingly favored due to their convenient recoverability and strong repeatability [9,10,11,12], and include metallic oxide, MOFs, etc. [13,14]. Meanwhile, MOFs, as a kind of novel porous metal-organic framework material, due to their high specific surface area, large porosity, and good structural stability have shown wide application prospects in gas adsorption [15,16,17], sensors [18,19,20], magnetic [21,22,23,24,25] optical materials [26,27,28,29,30,31,32], and catalysts [33,34,35,36], etc. Additionally, the MOF materials have been utilized to perform photocatalytic hydrogen production [37,38,39,40,41] and carbon dioxide reduction reactions [42,43,44,45]. For example, Su reported a amine-functionalized Co(II)-MOF catalyst through a one-pot solvothermal method. The material showed good photocatalytic hydrogen evolution activity (1102 μmol g^−1^ h^−1^. In addition, the CO_2_ reduction performance was also explored, in which -NH_2_ was used for light absorption and cobalt-oxygen clusters as catalytic nodes [46]. Besides, Du synthesized Ni-MOF materials displaying high catalytic activity for visible-light-driven hydrogen production under the illumination of white LED or direct sunlight [47]. Additionally, the heterogeneous nature and recyclability of the catalyst were studied carefully.

The research about MOF catalysts for hydrogen generation has made tremendous advances, including utilizing some organic ligands containing photosensitive groups, such as -NH_2_- and so on. To our knowledge, the MOFs catalytic splitting decomposition of water into hydrogen generally consists of three parts: a photosensitizer, co-catalyst, and sacrificial agent. In general, the photosensitizer and co-catalyst are mainly composed of a platinum complex, which usually costs a lot [48,49,50]. Therefore, developing a new class of MOFs material that does not need additional photosensitizers and co-catalysts under the catalytic hydrolysis process of hydrogen production is of great significance.

**Scheme 1:**
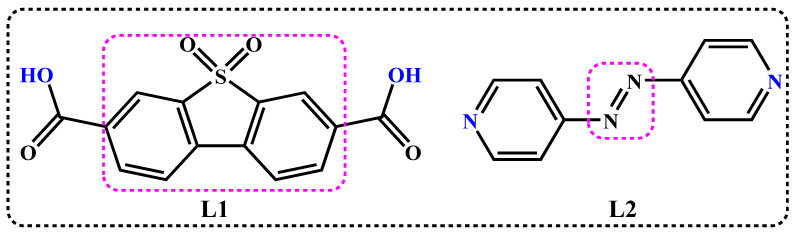
Chemical structures of 4, 4′-bibenzoic acid-2, 2′-sulfone (**L1**) and 4, 4′-azopyridine (**L2**) in this work.

Here, two organic ligands, 4, 4′-bibenzoic acid-2, 2′-sulfone (**L1**), 4, 4′-azopyridine (**L2**), were selected to build a new Zn-based metal-organic framework by coordinating with divalent zinc ion in a 1:1:1 ratio (Scheme 1). Finally, a new 3D MOF was obtained successfully in a high yield (63.5%), which was characterized by single-crystal X-ray diffraction, PXRD, IR, and elemental analysis, etc. Meanwhile, the PXRD tests after 1 was immersed in various solvents and pH conditions also reflected good solvent and pH stability of 1. Besides, the material showed high photocatalytic hydrogen production properties in pure water under the absence of the cocatalyst and photosensitizer. Furthermore, a comparative study of distinct solvent conditions, such as H_2_O and DMF/H_2_O in different ratios, displayed that the photocatalytic hydrogen generation properties reached their maximum when the ratio of DMF and H_2_O was 4:6. Additionally, the recyclability of the catalyst, as well as factors that affect the catalytic activity were investigated and described in detail.

## 2. Results

### 2.1. The Structure of Complex ***1***

**Figure 1:**
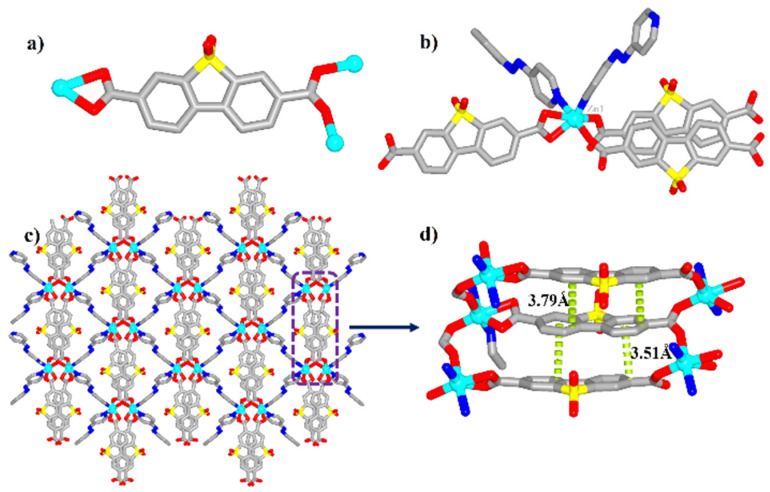
Single-crystal X-ray structure of **1**. Coordination geometry of ligand **L1** (**a**); coordination environment of Zn^2+^ ions in **1** (**b**); view of the 3D framework of **1** (**c**); view of π···π stacking interaction (3.79 Å and 3.51 Å) between **L1** ligands (**d**).

The Zn (II) site in **1** was six-coordinated by four carboxylic oxygen atoms from three **L1** ligands, together with two nitrogen atoms belonging to two same **L2** ligands. Thereinto, the two carboxylic acid groups of **L1** ligand were coordinated by different coordination modes, viz., chelating coordination and monotone coordination form, to link three zinc ions. In organic ligand **L2**, the azo bonds existed in a stable *trans* configuration. The bond lengths were Zn1-O3 = 2.006 (2) Å, Zn1-O4 = 2.169 (2) Å, Zn1-O5 = 2.357 (3) Å, Zn1-O6 = 2.070 (2) Å, Zn1-N1 = 2.112 (3) Å, and Zn1-N4 = 2.194 (3) Å (Figure 1a). The adjacent Zn···Zn distances bridged by **L1** and **L2** were 5.068 and 13.224 Å, respectively. Interestingly, there existed obvious π···π stacking interaction between the opposite **L1** ligands (3.51 Å). Therefore, the coordination mode of Zn^2+^ ions and accumulation effect resulted in the formation of a three-dimensional (3D) framework (Figure 1).

### 2.2. The TGA and Stability Exploration of ***1***

**Figure 2:**
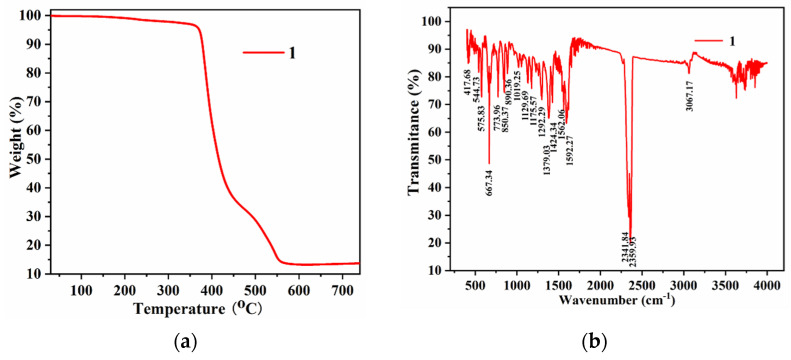
Thermogravimetric analysis curve of **1** (**a**); IR spectrum of complex **1** (**b**).

Elemental analysis (EA), thermogravimetric (TG) analysis, Fourier transform infrared (FT-IR) spectrum, and powder X-ray diffraction (PXRD) were performed to characterize MOF complex **1**. The TG curve showed that the initial 3.5% weight loss of **1** happened at 150–360 °C, which should be ascribed to the loss of a small number of free water and DMF molecules. Then, a large weight loss (67.4%) could be observed from 360 to 485 °C, which was consistent with the weight of ligand **L1** (calcd. 67.6%), reflecting the decomposition of the skeleton of compound **1** and the loss of **L1**. After that, the residual components of **1** were gradually lost and eventually converted to zinc oxides, nitrides, and sulfides. This result is consistent with those of EA and crystal data (Figure 2). In addition, the IR spectrum of complex **1** exhibited a strong band at 1180 cm^−1^ owing to the C-N stretching vibrations and strong absorptions at 1592 cm^−1^ and 1302 cm^−1^ due to stretch of the C=O and C-O bonds from the carboxylate group of **L1** (Figure 2b). Moreover, the sharp strong absorptions at 1424 cm^−1^ and 1379 cm^−1^ are attributable to the stretching vibrations of N=N and C=S bonds. Additionally, the C-H bond absorption peaks of the pyridine group in **L2** could be observed at 3067 cm^−1^.

**Figure 3:**
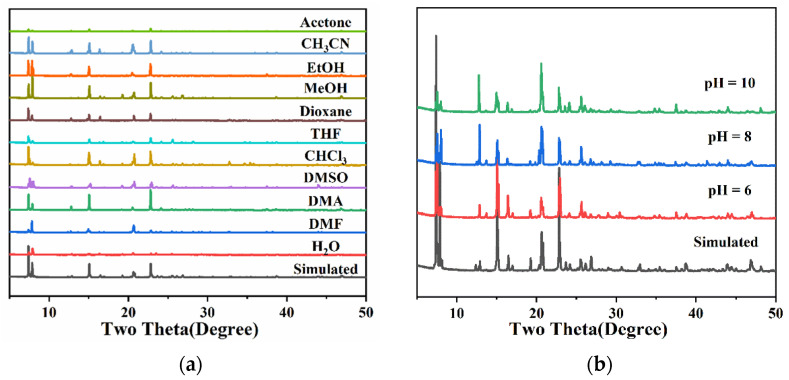
PXRD patterns of simulated (black) and after being immersed samples in different solvents of **1** (**a**); PXRD patterns of simulated (black) and after being immersed samples under different pH conditions of **1** (**b**).

In addition, the PXRD pattern showed that the diffraction peaks of as-synthesized Zn-MOF matched well with the simulated one in spite of the difference in peak intensity, suggesting the as-synthesized sample was single phase. Besides, a wider range of structural stability is significant for the study of the properties of the material in different conditions. Therefore, these samples were immersed in different solvents, such as CH_3_CN, EtOH, MeOH, Dioxane, DMSO, CHCl_3_, and so on for 3 days. Meanwhile, other samples were soaked in different pH solvents (range from 3–12). After that, the PXRD tests were carried out. The result displayed the PXRD matching was consistent between these samples and simulated data. Besides, the Zn leaching after being soaked in different pH solutions (pH = 3, 4, 6, 8, 10, 12) for 3 days was confirmed to be 63.84%, 60.46%, 4.72%, 4.86%, 5.28%, and 44.37% by inductively coupled plasma optical emission spectrometry (ICP-OES). The high Zn leaching reflected that the structures of complexes **1** in pH 3, 4, and 12 solution were destroyed. Additionally, the low Zn leaching value reflected the good stability of complexes **1** in the solutions (pH = 6, 8, 10) (Figure 3a). The results suggested good solvents and pH stability (range from 6–10) of **1**.

### 2.3. The UV-Vis Absorption and Fluorescence Emission Spectra

**Figure 4:**
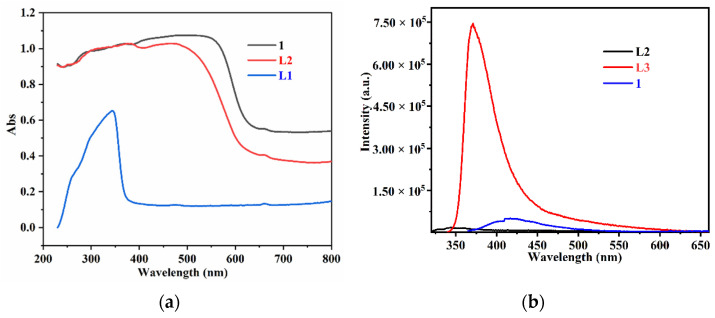
Solid-state UV-Vis absorption; (**a**) emission spectra (**b**) of **L1**, **L2**, and **1**.

To characterize the optical properties of MOF **1**, the solid-state UV-vis absorption spectra of ligands **L1** and **L2**, and **1** were measured and compared under ambient conditions. The results clearly showed that ligand **L2** displayed a broad absorption ranging from 250 to 500 nm. While, the complex also displayed a stronger and broader absorption under same variation range (from 250 to 550 nm). However, the pristine **L1** only exhibited sharp absorption ranging from 250 to 350 nm (Figure 4a). Therefore, compared with the pristine ligand **L1** and **L2**, the adsorption bands of **1** exhibited a significant red-shift. These properties are likely related to the effect of coordinated Zn (II) ions on the excited state of the ligand, d-d transitions of Zn (II) ions, and π···π* stacking interaction of between **L1** ligands [51,52]. Besides, the Kubelka–Munk representation of **1** was also studied (Figure 4a). The band-gap energy (Eg) of **1** determined from Tauc’s plot was 1.96 eV (Figure S7). The result reflected that **1** could be taken into account as ideal semiconductive MOFs and that such low energy values are infrequent for the MOF-series materials [53,54]. The result was very meaningful for photocatalytic hydrolysis to produce hydrogen because the valence band absorbs light energy to excite electrons into the conduction band, resulting in the production of light electrons. The electrons could be used to reduce water to produce hydrogen.

Meanwhile, the solid-state fluorescence emission spectra of ligands **L1** and **L2** and compound **1** were also explored carefully by the steady-state transient fluorescence spectrometer. By observing the data carefully, ligand **L1** showed very strong blue fluorescence emission at 375 nm when excited at 307 nm. However, an extremely weak blue fluorescence emission at 350 nm could be observed for ligand **L2** when excited at its optimum excitation wavelength of 326 nm. Besides, another weak fluorescence emission was also found at 430 nm when the excitation wavelength was at 350 nm, which was attributed to the signal of complex **1**. In comparison to pristine ligand **L1**, the dramatic reduction in emission intensity for **L1** ligand in **1** could be attributed to the fact that **L2** ligand has strong absorption in the infrared region, and the large overlaps between the absorption peak of ligand **L2** and the emission peak of ligand **L1** in complex **1** led to the weak fluorescence emission of complex **1** (Figure 4b). In addition, the azo group is a chromophore that can absorb visible light of a certain wavelength, resulting in **1** having a strong photogenic electron effect. To evaluate the photostability of Zn-MOF, we carried out cycle experiments.

### 2.4. The Photocurrent Response Performance of Complex ***1***

**Figure 5:**
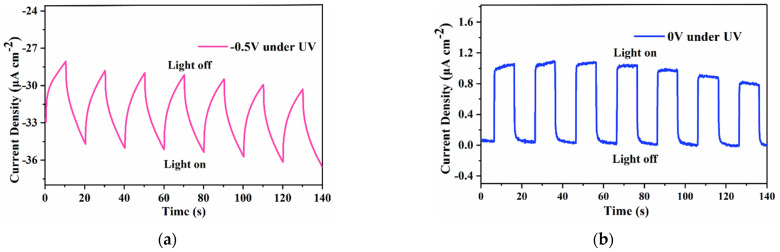
Transient photocurrent/time profiles of the bare glassy carbon electrode and **1**, which were coated on the glassy carbon electrode in a KCl electrolyte (0.1 mol·L^−1^) at a bias of −0.5 V (**a**) and 0.0 V (**b**), with and without light irradiation.

In order to explore the photoelectric performance of complex **1**, we evaluated the photoelectron response performance of this luminescent material. Firstly, the MOF powder-modified indium tin oxide (ITO) glass was chosen to act as the working electrode, and then the working electrode was tested in a sodium sulfate aqueous solution in a standard three-electrode system. Transient photocurrent/time curves for a few of on–off cycles under Xe lamp irradiation reflected that the electrode modified by complex **1** provided a detailed process about their charge separation efficiency levels (Figure 5). The results showed that when the voltage was at 0 V, once the lamp was turned on, the photocurrent value increased quickly to a certain value (1.0 μA·cm^–2^), and when the irradiation was canceled, the photocurrent value descended rapidly, and these results were found for three repeats of the experiments (Figure 5b). Besides, when the experiment was performed at −0.5 V, the current value could reach 28.5 μA·cm^–2^, and once the lamp was turned on, the photocurrent value increased rapidly to a very high value (34.5 μA·cm^–2^) (Figure 5a). These dramatical photocurrent responses evidenced higher separation efficiency levels for photogenerated electron–hole pairs.

### 2.5. The Visible-Light-Driven H_2_ Production

**Figure 6:**
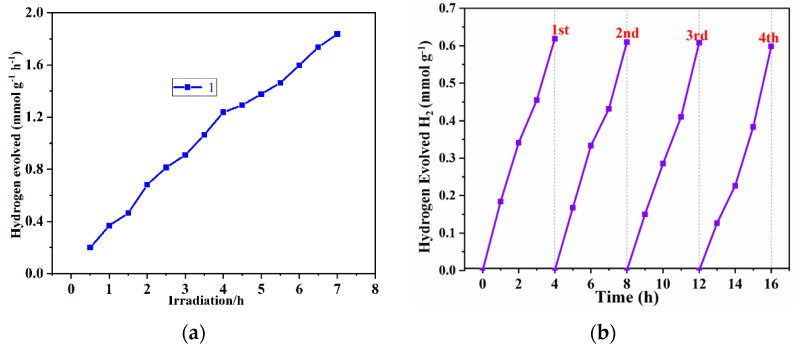
(**a**) The rate of hydrogen photogenerated over catalyst **1** (80 mg) in aqueous solutions (100 mL, pH = 11.5) containing Na_2_S and Na_2_SO_3_ under visible light irradiation; (**b**) durability testing for the hydrogen generation of **1**, which was recycled from the reaction solution and used again under the same catalytic condition three times.

Based on MOF catalyst **1**, a series of visible-light-driven H_2_ generation experiments were executed seriously in a H_2_O solution and a mixture solution of DMF and H_2_O at the surrounding environment accompanied by Na_2_S and Na_2_SO_3_ as the sacrificial agents. Meanwhile, no additional photosensitizers and co-catalysts were utilized in the process. The study result reflected that under optimized conditions, **1** is highly active for the photocatalytic decomposition of water molecules, generating the production of hydrogen molecules, as well as an average rate of approximately 261 μmol g^−1^ h^−1^ (Figure 6a). Additionally, a new experiment was performed in the absence of **1** under the measured conditions, and the result showed that no detectable hydrogen was found, reflecting that complex **1** played a vital role in the course of photocatalytic hydrogen generation from the water solution. Importantly, the high catalytic efficiency of **1** for decomposing water molecules to generate hydrogen indicated the excellent potential advantage of **1** with no need for additional photosensitizers and cocatalysts in the course of photocatalytic experment. Once the photocatalyzed reaction was finished, the catalyst could be recovered by centrifugation and the complex was washed with ethanol. Then, the recovered catalyst was added into another fresh solution of Na_2_S and Na_2_SO_3_ and illuminated again, and hydrogen could be reproduced with a similar rate as before. Under the same treatment, the catalyst could be used repeatedly for four catalytic cycles (Figure 6b). The PXRD patterns (Figure S2) indicated that **1** could retain its structural stability after the photocatalytic reaction. These results highlight that **1** is an active photocatalyst for visible-light-driven hydrogen evolution. According to the X-ray photoelectron spectra (XPS, Figure S5) of as-synthesized Zn-MOF, the peaks of Zn 2p 3/2 (1022.0 eV) and Zn 2p 1/2 (1045.1 eV) were all sharp and symmetrical, demonstrating that the valence of Zn was +2. In addition, for the Zn-MOF after the photocatalytic reaction, The XPS revealed shifts of 0.4 eV in the Zn 2p3/2 (1021.6 eV) and Zn 2p1/2 (1044.7 eV) peaks toward shift-binding energies (Figure S6). The reason for the lower BE (i.e., less negative orbital energy) of the Zn cationic from the Zn framework can be envisioned, qualitatively, as originating from the less positive charge of the Zn cationic, possibly because the sacrificial agents Na_2_S and Na_2_SO_3_ provided electrons to fill the holes of the Zn cationic in the photocatalytic process.

The apparent quantum yield (AQY) value for Zn-MOF was calculated to be 0.5% by using a Xe lamp equipped with a 420-nm monochromatic light filter and calculated via the following equation:

$$\mathrm{AQY}(\%)=\frac{2\times {\;\mathrm{number}\;\mathrm{of}\;H}_{2}}{\mathrm{number}\;\mathrm{of}\;\mathrm{incident}\;\mathrm{photons}}$$

**Figure 7:**
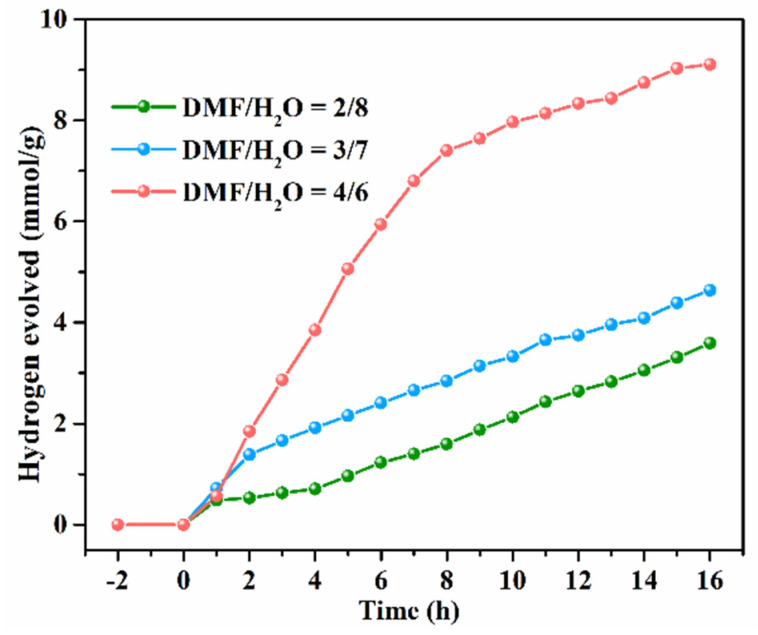
The rate of hydrogen photogenerated over catalyst **1** (80 mg) in different solvent ratio (Total volume: 100 mL, pH = 11.5) containing Na_2_S and Na_2_SO_3_ under visible light irradiation.

As we know, under the absence of PS and co-catalysts, the catalyst and sacrificial agent were very important for the production of hydrogen molecules, and changing the individual variables to optimize the photocatalytic activity of **1** is very significative. In addition, previous literature has shown that the types of another solvent would also affect the activity on producing hydrogen. Therefore, different mixture solutions of DMF/H_2_O, MeCN/H_2_O, DMAc/H_2_O, isopropanol/H_2_O, and DMSO/H_2_O were discussed. The results revealed that the DMF/H_2_O solutions had higher amounts of hydrogen under the same conditions. Moreover, the effect of different ratios of DMF/H_2_O on the influence of photocatalytic activity were also carefully explored [46]. Thus, a series of experimental tests were performed carefully and the reaction results acquired in different solvent ratios of DMF/H_2_O were displayed and compared (Figure 7). The hydrogen amount produced from these experimental results showed a very similar variation tendency, namely, increasing quickly in the first hours but gradually descending and eventually reaching the maximum. At the same time, we found that different solvent ratios of DMF/H_2_O would also affect the photocatalytic activity on the generation of hydrogen in this MOF structure, for example, in DMF/H_2_O, when the ratio varied from 2/8 to 3/7 (mL/mL), an obvious enhancement could be found about the average activity from 224 to 291 μmol g^−1^ h^−1^. Then, when the ratio reached the ratio of 6/4, two distinct hydrogen production rates were found. In the first 8 h, the average rate was very high and the value reached 743 μmol g^−1^ h^−1^. Then, the rate reduced gradually and the value of average activity was about 396 μmol g^−1^ h^−1^ in the next eight hours. Totally, over 16 h, the experiment performed in DMF/H_2_O (*v*/*v*, 4/6) produced the most amount of hydrogen (9.1 mmol/g), while the one in DMF/H_2_O (*v*/*v*, 2/8) produced a lower amount (3.59 mmol). The influence of all these factors on catalytic activity should be ascribed to the effect on light absorption and electron transition during the photocatalytic process.

### 2.6. The Photocatalytic Mechanism Study of Hydrogen Production

**Figure 8:**
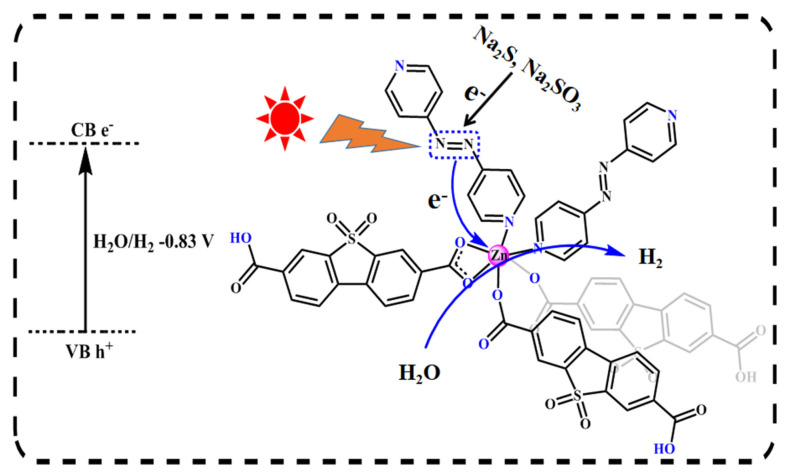
Proposed photocatalytic mechanism of hydrogen evolution for Zn-MOF under visible-light irradiation.

Careful study revealed that the photocatalytic activity is attributable to the electron transfer between the ligand and the metal [55,56,57,58,59,60,61,62,63,64,65,66,67]. Then, the photocatalytic mechanism for hydrogen evolution could be described as follows (Figure 8). The azo unit is a good chromophore and could effectively absorb photoelectrons. Under visible illumination, the azo unit absorbed photoelectrons as well as changed from the ground state to the excited state. Then, the electrons were transferred to Zn clusters. Subsequently, H_2_O molecules acquired electrons from the Zn clusters and caused the production of hydrogen. The catalytic circle was fulfilled. Due to the rapid recombination of CB electrons and VB holes in the photocatalytic reaction process, it is difficult to achieve efficient hydrogen production by only using a photocatalyst in pure water. Therefore, the addition of an electron-sacrificing agent can make the irreversible photogenerated hole reaction caused by the valence band, thus accelerating the photogenerated charge separation. Since the sacrificial agent is always consumed in the reaction process, electron donors need to be continuously added into the system to maintain efficient hydrogen production reaction.

## 3. Experiment

### 3.1. Materials and Methods

Analytically pure Zn(NO_3_)_2_·6H_2_O was used as metal salt, and two ligands, 4, 4′- bibenzoic acid-2, 2′-sulfone (**L1**) and 4,4′-azopyridine (**L2**), were chosen as connectors. In addition, the Na_2_S and Na_2_SO_3_ were selected as sacrificial agents, and these chemicals were purchased from the chemical company and used without further purification. In addition, the Infrared spectra (IR) measurement was performed on a Nicolet 170SX spectrometer in the range of 4000–400 cm^–1^. Meanwhile, the Elemental analyses of C, H, and N were executed on a model 2400 Perkin-Elmer analyzer. Besides, a series of PXRD tests reflecting the structural stability were collected on a Bruker D8-ADVANCE X-ray diffractometer with Cu Kα radiation (λ = 1.5418 Å), including a 2θ range of 5°–50° at r.t (room temperature) with a step of 0.02° (2θ) and a counting time of 0.2 s/step. The Thermogravimetric analysis (TGA) experiments were carried out utilizing the SII EXSTAR6000 TG/DTA6300 thermal analyzer from 25 to 800 °C under nitrogen protection as well as a heating rate of 10 °C min^−1^. Photoelectric measurements were acquired with a CHI 660E electrochemical workstation, the working area of the working electrode was 1.0 cm^2^, and the MOF was modified by ITO. Moreover, the Ag/AgCl was used as a reference electrode and platinum-wire electrode as a counter electrode. All electrochemical tests were performed at room temperature in 0.5 M Na_2_SO_4_ solution. The leaching test was carried out on the Ultima inductively coupled plasma OES spectrometer (ICP-OES).

General procedure for photocatalytic H_2_ evolution. The photocatalytic hydrogen evolution experiments were carried out under a 300 W Xe lamp (PLS-SXE 300C, Beijing PerfectLight Co., Ltd., Beijing, China), and the reactor was a top-irradiation-type Pyrex reaction cell connected to a closed-gas circulation and evacuation system. In a typical experiment, 80 mg of catalyst was dispersed in 100 mL of an aqueous solution containing Na_2_S (35.0 mmol, 8.4 g) and Na_2_SO_3_ (25.0 mmol, 3.15 g) as the sacrificial electron donors. Afterwards, the reaction system was carefully sealed before evacuation for 15 min to completely remove air. Finally, the sealed quartz reactor was irradiated by a Xe lamp equipped with an optical cut off filter (λ > 420 nm) at a fixed distance (10 cm). During the photocatalytic reaction, the reaction solution was continuously magnetically stirred and kept at 15 °C by a flow of cooling water. The evolved gases were analyzed by gas chromatography with a thermal conductive detector (TCD) and a 5 Å molecular sieve column using N_2_ as the carrier gas.

X-ray Photoelectron Spectroscopy (XPS). XPS was conducted using a PHI 5300 spectrometer with a Perkin-Elmer Dual Anode X-ray source operating with magnesium radiation with monochromatic Mg Kα radiation (hν 1253.6 eV) at 13 kV and 250 W and a pass energy of 17.9 eV. A step size of 0.025 eV was used, and 180 sweeps were averaged. Emitted photoelectrons were detected by a hemispherical analyzer and the operating pressure in the sampling chamber was below 1 × 10^−7^ Torr. The spectral scanning range for nitrogen 1s was 410−390 eV and for cobalt 2p was 765−815 eV. The spectra were calibrated according to the C 1s peak, which is known to occur at 284.6 eV.

### 3.2. Synthesis of Complex ***1***

A mixture of metal salt Zn(NO_3_)_2_·6H_2_O (0.2 mmol, 0.058 g), organic connectors H_2_bps = 4, 4′-bibenzoic acid-2, 2′-sulfone (0.2 mmol, 0.061 g), and 4,4′-azopyridine (0.1 mmol, 0.018 g), reaction solvents N,N′-dimethylformamide (DMF, 4.5 mL), and H_2_O (2.5 mL) was placed in in a 20 mL screw-capped glass jar. Then, the mixture was sealed and heated at 118 °C for 3 days. After that, the reaction system was cooled to 30 °C with about a 6 °C per min cooling rate. After filtration and washing with an excess of N, N′-dimethylformamide (DMF), and ethanol, pink block crystals 1 were collected and dried at room temperature as a pure phase (Yield: 63.5% based on Zn). Anal. Calc. (%) for C_24_H_14_N_4_O_6_SZn: C 52.24, H 2.56, N 10.15; found (%): C 52.19, H 2.51, N 10.18. IR (KBr, cm^−1^): 3067 (w), 2359 (w), 1592 (s), 1562 (s), 1424 (s),1379 (s), 1292 (m), 1175 (m), 1129 (w), 1019 (w), 890 (m), 850 (m), 773 (w), 667 (m), 575 (w), 544 (w), 417 (w).

### 3.3. X-ray Crystallography

Single-crystal X-ray diffraction data for **1** were acquired at 298 K on an Oxford Diffraction SuperNova area-detector diffractometer by utilizing mirror optics monochromated Mo Kα radiation (λ = 0.71073 Å). CrysAlisPro [68] was utilized for the crystal data collection, general data reduction, and further empirical absorption correction. The crystal structure of **1** was solved by SHELXS-2014 and least-squares refined with SHELXL-2014 [69]. The crystal refinement parameters are provided in Table S2. The X-ray crystallographic coordinated for the structure reported in this study has been deposited at the CCDC under deposition number 2157013. These data can be obtained free of charge from the CCDC via http://www.ccdc.cam.ac.uk/Community/Requestastructure (accessed on 7 March 2022).

## 4. Conclusions

In summary, a new 3D zinc-based metal-organic framework 1 was built successfully based on a dicarboxylic acid and a dipyridine ligand. The structure and stability of complex **1** was explored in detail by single-crystal X-ray diffraction, IR, elemental analysis, and PXRD test, suggesting the good stability of **1** in different solvents and under distinct pH conditions. Besides, the visible-light-driven hydrogen generation was studied under the illumination of white LED in pure water or a mixture of DMF and H_2_O without additional photosensitizers and cocatalysts. The results showed that the visible-light-driven hydrogen evolution ability was enhanced when the ratio of DMF and H_2_O varied from 2/8 (mL/mL) to 4/6, and the activity enhanced from 224 to 743 μmol g^−1^ h^−1^) gradually, reflecting that H_2_O content in solvents could affect the activity on producing hydrogen. In addition, the strong absorption of **1** in the UV-vis region with narrow band gap energies (energy: 1.96 eV) reflected good semiconductor performance. The self-sensitized complex **1** opens a new direction of future development of low-cost photocatalysts for efficient and long-term solar fuel production.

## Data Availability

Not applicable.

## Supplementary Materials

The following supporting information can be downloaded at: https://www.mdpi.com/article/10.3390/molecules27061917/s1, Figure S1: PXRD patterns of simulated (Red) and after photocatalytic test in H_2_O (Black) of **1**; Figure S2: PXRD patterns of simulated (yellow) and after being immersed samples under H_2_O solvent or different mixed solutions of H_2_O and DMF of **1**; Figure S3: PXRD patterns of simulated (Black) and after photocatalytic test in a ratio 6:4 of H_2_O and DMF (Blue) of **1**; Figure S4: The fluorescence emission spectra before and after the photocatalytic reaction of 1 in aqueous solution; Figure S5: XPS results of **1** (as synthesized), including (a) complete and (b) Zn 2p spectra; Figure S6: XPS results of **1** (after photocatalytic reaction), including (a) complete and (b) Zn 2p spectra; Figure S7: The Tauc’s plot (αhv)1/2 vs. (hv), where α is absorbance based on solid-state UV-Vis absorption in Figure 4a. Table S1: Photocatalytic activity of some typical MOF-based photocatalysts for the hydrogen evolution reaction. Table S2: Crystal data and structure refinement for complex **1**.
