# Supplementary material for: Stable Zinc-Based Metal-Organic Framework Photocatalyst for Effective Visible-Light-Driven Hydrogen Production
Source: Molecules. 2022 Mar 16;27(6):1917. doi: 10.3390/molecules27061917 (PMC8952245; doi:10.3390/molecules27061917)
Supplement: Supplementary file 1 [file molecules-27-01917-s001.zip › molecules-1627064-supplementary.pdf]

Supporting information for

Stable Zinc-Based Metal-Organic Framework Photocatalyst for Effective Visible-Light-Driven Hydrogen Production

Li-Long Dang [1,3], Ting-Ting Zhang [1,2], Ting-Ting Li [1], Tian Chen [1], Ying Zhao [1], Chen-Chen Zhao [1] and Lu-Fang Ma [1,*]

[1] Henan Province Function-Oriented Porous Materials Key Laboratory, College of Chemistry and Chemical Engineering, Luoyang Normal University, Luoyang 471934, China; danglilong8@163.com (L.-L.D.); zhangtingting131@126.com (T.-T.Z.); tingtinglichem@163.com (T.-T.L.); ctian2022@163.com (T.C.); zhaoyingchem@126.com (Y.Z.); chenchenzhao2022@163.com (C.-C.Z.)

[2] College of Materials and Chemical Engineering, China Three Gorges University, Yichang 443002, China

[3] Shanghai Key Laboratory of Molecular Catalysis and Innovative Materials, Fudan University, Shanghai 200438, China

* Correspondence: mazhuxp@126.com

A. Experimental Section

1. Materials and Methods

B. Supporting Figures

Figure S1. PXRD patterns of simulated (Red) and after photocatalytic test in H_2O (Black) of 1.

Figure S2. PXRD patterns of simulated (yellow) and after being immersed samples under H_2O solvent or different mixed solutions of H_2O and DMF of 1.

Figure S3. PXRD patterns of simulated (Black) and after photocatalytic test in a ratio 6:4 of H_2O and DMF (Blue) of 1.

Figure S4. The fluorescence emission spectra before and after the photocatalytic reaction of 1 in aqueous solution.

Figure S5. XPS results of 1 (as synthesized)

Figure S6. XPS results of 1 (after photocatalytic reaction)

Figure S7. The Tauc's plot $(\alpha hv)^{1/2}$ vs (hv), where α is absorbance

C. Supporting Tables

Table S1. Photocatalytic activity of some typical MOF-based photocatalysts for the hydrogen evolution reaction.

Table S2. Crystal data and structure refinement for complex 1

D. Supporting References

A. Experimental Section

1. Materials and Methods

Analytically pure $Zn(NO_3)_2 \cdot 6H_2O$, 4, 4'- bibenzoic acid-2, 2'-sulfone (**L1**), 4,4'-azopyridine (**L2**) and Na_2S, Na_2SO_3, Na_2SO_4 were purchased from drug companies and used without further purification process. In addition, the Infrared spectra (IR) were performed on the Nicolet 170SX spectrometer in the 4000–400 cm^{-1}. Meanwhile, the Elemental analyses tests of C, H, N were performed carefully on an instrument of the model 2400 Perkin-Elmer analyser. A series of PXRD experiments were carried out on a Bruker D8-ADVANCE X-ray diffractometer with Cu Kα radiation (λ = 1.5418 Å). Measurements were made in a 2θ range of 5−50° at room temperature with a step of 0.02° (2θ) and a counting time of 0.2 s/ step, and the operating power was 40 KV, 40 mA. Thermogravimetric analysis (TGA) experiments were executed utilizing SII EXSTAR6000 TG/DTA6300 thermal analyzer from 25 to 800 °C under a nitrogen atmosphere as well as a heating rate of 10 °C min^{-1}. Photoelectric measurements were acquired with a CHI 660E electrochemical workstation, the working area of working electrode is 1.0 cm^2, and the MOF modified by ITO. Moreover, the Ag/AgCl was used as a reference electrode and platinum wire electrode as a counter electrode. All electrochemical tests were performed at room temperature in 0.5 M Na_2SO_4 solution.

B. Supporting Figures

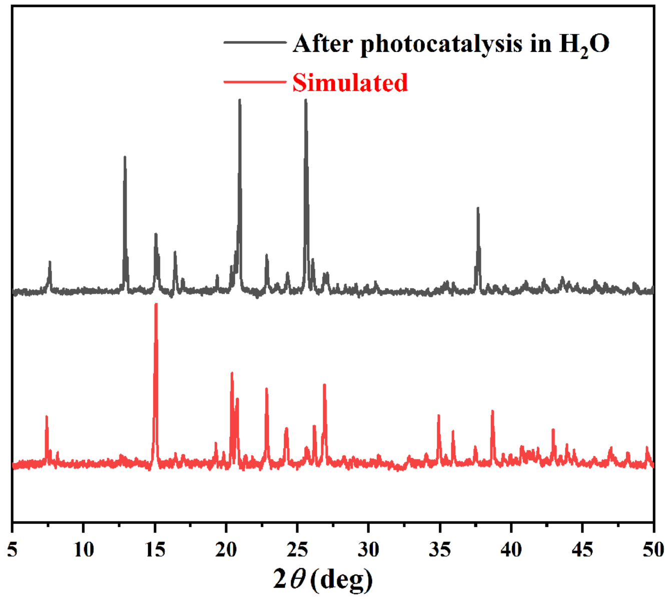

Figure S1. PXRD patterns of simulated (Red) and after photocatalytic test in H_2O (Black) of **1**.

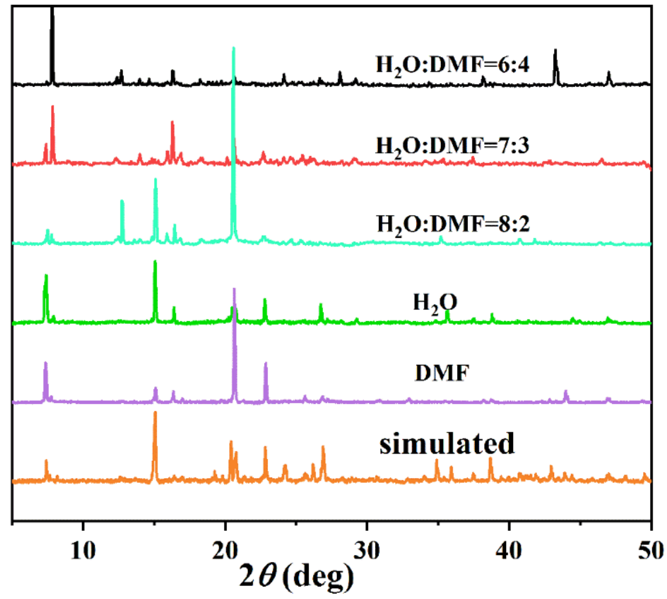

Figure S2. PXRD patterns of simulated (yellow) and after being immersed samples under H_2O solvent or different mixed solutions of H_2O and DMF of **1**.

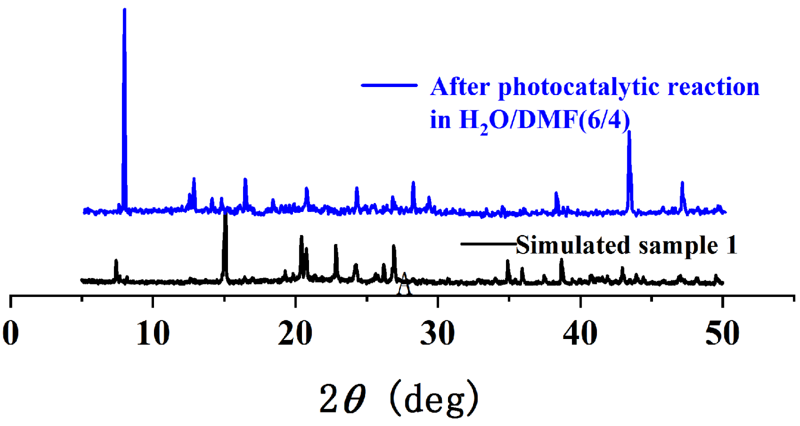

Figure S3. PXRD patterns of simulated (Black) and after photocatalytic test in a ratio 6:4 of H_2O and DMF (Blue) of **1**.

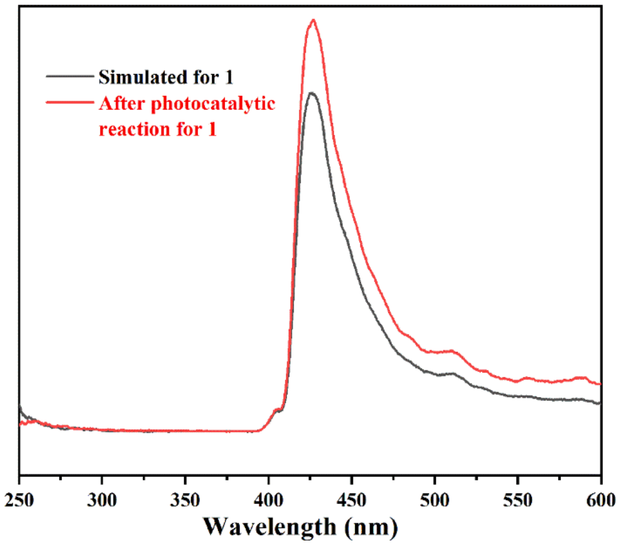

Figure S4. The fluorescence emission spectra before and after the photocatalytic reaction of **1** in aqueous solution.

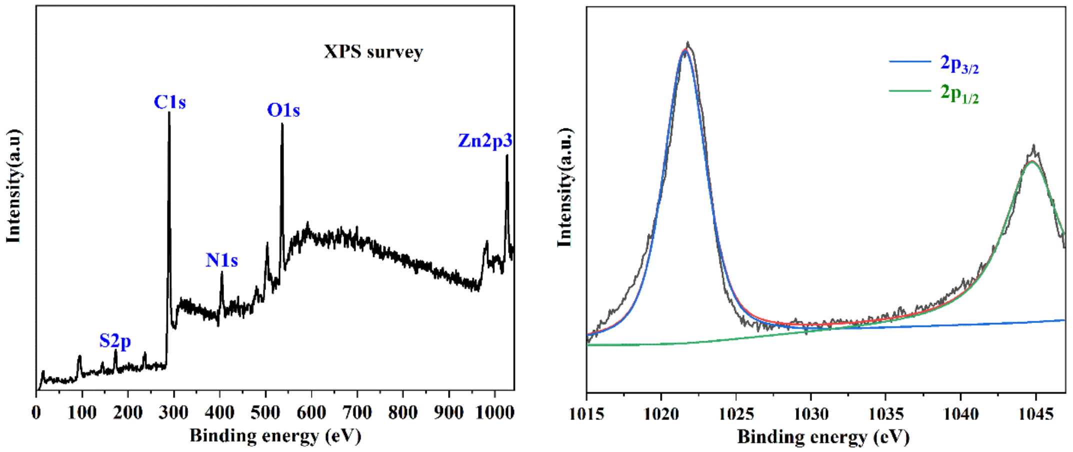

(**a**) (**b**)

Figure S5. XPS results of **1** (as synthesized), including (**a**) complete and (**b**) Zn 2p spectra.

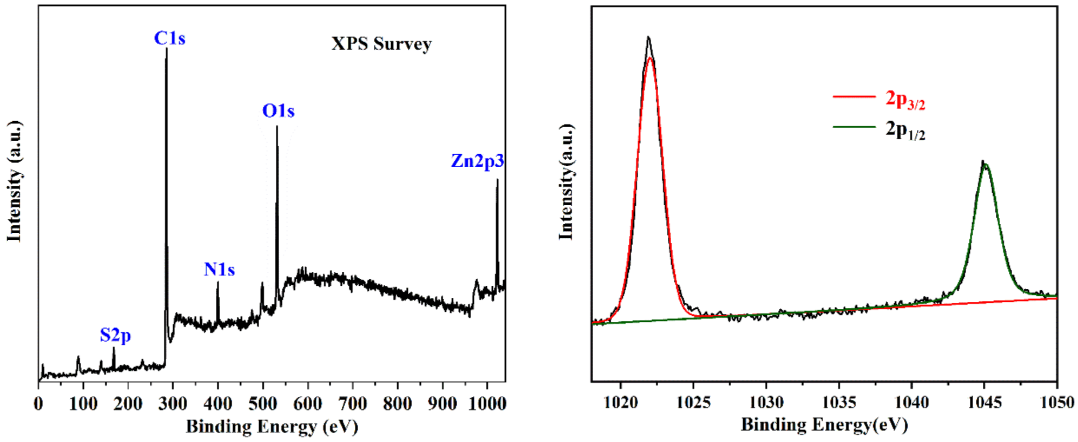

(**a**) (**b**)

Figure S6. XPS results of **1** (after photocatalytic reaction), including (**a**) complete and (**b**) Zn 2p spectra.

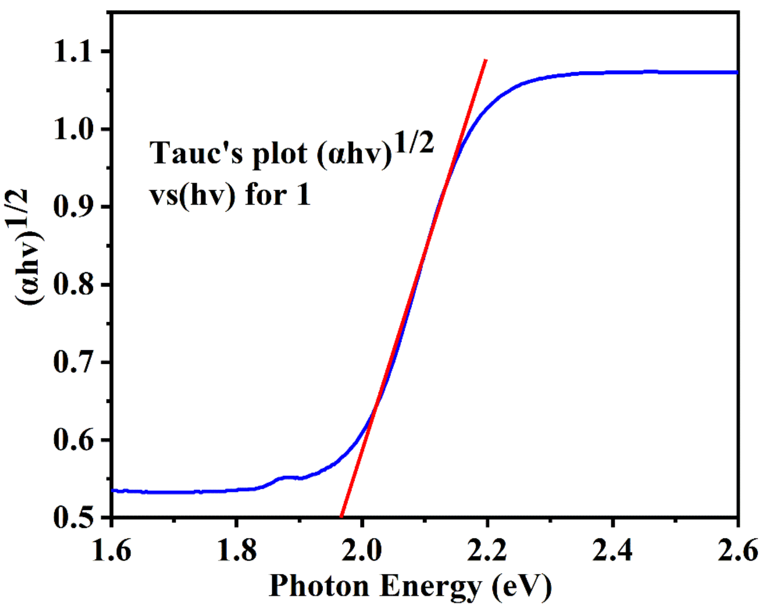

Figure S7. The Tauc’s plot $(\alpha h\nu)^{1/2}$ vs (hv), where α is absorbance based on solid-state UV-Vis absorption in Figure 4a.

C. Supporting Tables

Table S1. Photocatalytic activity of some typical MOF-based photocatalysts for the hydrogen evolution reaction.

Photocatalyst	Sacrificial reagent	Production rate (μmol h^{-1} g^{-1})	Ref.
Ti-MOF	Triethanolamine (TEOA)	0	[1]
Ti-MOF-NH_2	Triethanolamine (TEOA)	≈170	[1]
0.5 wt.% Pt/Ti-MOF-NH_2	Triethanolamine (TEOA)	≈330	[1]
1.5wt.%Pt/Ti-MOF-NH_2	Triethanolamine (TEOA)	≈500	[1]
2 wt.% Pt/Ti-MOF-NH_2	Triethanolamine (TEOA)	≈460	[1]
Small-sized Ni NPs anchored in MOF-5	Triethanolamine (TEOA)	3,022	[2]
Ni@MOF-5	Triethanolamine (TEOA)	30220	[2]
Pt complex immobilized MOF-253	CH_3CN	≈58,000	[3]
UiO-66	Na_2S, Na_2SO_3	0	[4]
UiO-66/CdS	Na_2S, Na_2SO_3	1,700	[4]
ErB dye-sensitized Pt/UiO-66 octahedrons	Methanol	460	[5]
Ti-MOF-Ru(tpy)$_2$	Triethanolamine (TEOA)	≈200	[6]
Pt@UiO-66	Triethanolamine (TEOA)	3.9	[7]
Co-MOF	Triethanolamine (TEOA)	1102	[8]
Cu_2I_2-based MOF	Triethanolamine (TEA)	7090	[9]
NiS/$Zn_{0.5}Cd_{0.5}S$	Na_2S, Na_2SO_3	16780	[10]
Pt@NH_2-UiO-66	Triethanolamine (TEOA)	257.38	[11]
UCNPs-Pt@MOF/Au	Triethanolamine (TEOA)	280	[12]
Al-TCPP-Pt	Triethanolamine (TEOA)	129	[13]
MIL-101(Cr)@Co	Triethanolamine (TEOA)	1500	[14]
Ru-TBP-Zn	Triethanolamine (TEOA)	240	[15]
PCN-415-NH_2	Triethanolamine (TEOA)	594	[16]
[Cu_2I_2(BPEA)](DMF)$_4$	Triethanolamine (TEA)	4220	[17]
Zn-MOF	Na_2S, Na_2SO_3	743	This work

Table S2. Crystal data and structure refinement for complex 1

Empirical formula	$C_{24}H_{14}N_4O_6SZn$	
Formula weight	551.82	
Temperature	275.57 K	
Wavelength	1.34138 Å	
Crystal system	Monoclinic	
Space group	*Cc*	
Unit cell dimensions	a = 14.7101(6) Å	α= 90°.
	b = 21.8583(9) Å	β= 111.9830(10)°.
	c = 7.4882(3) Å	γ= 90°.
Volume	2232.68(16) $Å^3$	
Z	4	
Density (calculated)	1.642 Mg/m^3	
Absorption coefficient	1.799 mm^{-1}	
F(000)	1120.0	
Crystal size	0.15 × 0.12 × 0.11 mm^3	
Radiation	GaKα (λ = 1.34138)	
Theta range for data collection	6.646 to 146.656°.	
Index ranges	-21<=h<=21, -31<=k<=31, -10<=l<=8	
Reflections collected	16215	
Independent reflections	5719 [R(int) = 0.0437]	
Max. and min. transmission	0.751 and 0.421	
Refinement method	Full-matrix least-squares on F^2	
Data / restraints / parameters	5719/2/325	
Goodness-of-fit on F^2	1.088	
Final R indices [I>2sigma(I)]	R1 = 0.0423, wR2 = 0.1131	
R indices (all data)	R1 = 0.0436, wR2 = 0.1190	
Largest diff. peak and hole	1.01 and -0.50 e.$Å^{-3}$	

D. Supporting References

1. Toyao, T.; Saito, M.; Horiuchi, Y.; Mochizuki, K.; Iwata, M; Higashimura, H.; Matsuoka, M. Efficient hydrogen production and photocatalytic reduction of nitrobenzene over a visible-light-responsive metal−organic framework photocatalyst. *Catal. Sci. Technol.* **2013**, *3*, 2092–2097.
2. Zhen, W.; Ma, J.T.; Lu, G.X. Small-sized Ni (1 1 1) particles in metal-organic frameworks with low over-potential for visible photocatalytic hydrogen generation. *Appl. Catal. B Environ.* **2016**, *190*, 12–25.
3. Zhou, T.; Du, Y.; Borgna, A.; Hong, J.; Wang, Y.; Han, J.; Zhang, W.; Xu, R. Post-synthesis modification of a metal−organic framework to construct a bifunctional photocatalyst for hydrogen production. *Energy Environ. Sci.* **2013**, *6*, 3229–3234.
4. Lin, R.; Shen, L.; Ren, Z.; Wu,W.; Tan, Y.; Fu, H.; Zhang, J.; Wu, L. Enhanced photocatalytic hydrogen production activity via dual modification of MOF and reduced graphene oxide on CdS. *Chem. Commun.* **2014**, *50*, 8533–8535.
5. Yuan, Y.; Yin, L.; Cao, S.; Xu, G.; Li, C.; Xue, C. Improving photocatalytic hydrogen production of metal–organic framework UiO-66 octahedrons by dye-sensitization. *Appl. Catal. B: Environ.* **2015**, *572*, 168–169.
6. Toyao, T.; Saito, M.; Dohshi, S.; Mochizuki, K.; Iwata, M.; Higashimura, H.; Horiuchi, Y.; Matsuoka, Y. Development of a Ru complex-incorporated MOF photocatalyst for hydrogen production under visible-light irradiation. *Chem. Commun.* **2014**, *50*, 6779–6781.
7. He, J.; Wang, J.; Chen, Y.; Zhang, J.; Duan, D.; Wang, Y.; Yan, Z. A dye-sensitized Pt@UiO-66(Zr) metal–organic framework for visible-light photocatalytic hydrogen production. *Chem. Commun.* **2014**, *50*, 7063–7066.
8. Liao, W.; Zhang, J.; Wang, Z.; Lu, Y.; Yin, S.; Wang, H.; Fan, Y.; Pan, M.; Su, C. Semiconductive Amine-Functionalized Co (II)-MOF for Visible-Light-Driven Hydrogen Evolution and CO2 Reduction. *Inorg. Chem.* **2018**, *57*, 11436–11442.
9. Shi, D.; Zheng, R.; Sun, M.; Cao, X.; Sun, C.; Cui, C.; Liu, C.; Zhao, J.; Du, M. Semiconductive Copper (I)-Organic Frameworks for Efficient Light-Driven Hydrogen Generation Without Additional Photosensitizers and Cocatalysts. *Angew. Chem. Int. Ed.* **2017**, *56*, 14637–14641.
10. Zhao, X.; Feng, J.; Liu, J.; Shi, W.; Yang,G.; Wang, G.; Cheng,P. An Efficient, Visible-Light-Driven, Hydrogen Evolution Catalyst NiS/ZnxCd1-xS Nanocrystal Derived from a Metal-Organic Framework. *Angew. Chem. Int. Ed.* **2018**, *26*, 9790–9794.
11. Xiao, J.; Shang, Q.; Xiong, Y.; Zhang, Q.; Luo, Y.; Yu, S.H.; Jiang, H.L. Boosting Photocatalytic Hydrogen Production of a Metal–Organic Framework Decorated with Platinum Nanoparticles: The Platinum Location Matters. *Angew. Chem. Int. Ed.* **2016**, *55*, 9389–9393.
12. Li, D,; Yu, S.; Jiang, H. From UV to Near-Infrared Light-Responsive Metal–Organic Framework Composites: Plasmon and Upconversion Enhanced Photocatalysis. *Adv. Mater.* **2018**, *30*, 1707377.
13. Fang, X.; Shang, Q.; Wang, Y.; Jiao,L.; Yao, T.; Li, Y.; Zhang, Q.; Luo, Y.; Jiang, H. Single Pt Atoms Confined into a Metal–Organic Framework for Efficient Photocatalysis. *Adv. Mater.* **2018**, *30*, 1705112.
14. Li, Z.; Xiao, J.; Jiang, H. Encapsulating a Co(II) Molecular Photocatalyst in Metal−Organic Framework for Visible-Light-Driven H2 Production: Boosting Catalytic Efficiency via Spatial Charge Separation. *ACS Catal.* **2016**, *6*, 5359–5365.
15. Lan, G.; Zhu, Y.; Veroneau, S.; Xu, Z.; Micheroni, D.; Lin, W. Electron Injection from Photoexcited Metal−Organic Framework Ligands to Ru2 Secondary Building Units for Visible-Light-Driven Hydrogen Evolution. *J. Am. Chem. Soc.* **2018**, *140*, 5326–5329.
16. Yuan, S.; Qin, J.; Xu, H.; Su, J.; Rossi, D.; Chen, Y.; Zhang, L.; Lollar, C.; Wang, Q.; Jiang, H.; Son, D.; Xu, H.; Huang, Z.; Zou, X.; Zhou,H. [Ti8Zr2O12(COO)16] Cluster: An Ideal Inorganic Building Unit for Photoactive Metal−Organic Frameworks. *ACS Cent. Sci.* **2018**, *4*, 105–111.

17. Shi, D.; Zheng, R.; Sun, M.; Cao, X.; Sun, C.; Cui, C.; Liu, C.; Zhao, J.; Du, M. Semiconductive Copper (I)-Organic Frameworks for Efficient Light-Driven Hydrogen Generation Without Additional Photosensitizers and Cocatalysts. *Angew. Chem. Int. Ed.* **2017**, *56*, 14637–14641.
